# Burnout and allostatic load among health workers engaged in human resourced-constrained hospitals in Accra, Ghana

**DOI:** 10.1186/s12913-022-08539-5

**Published:** 2022-09-14

**Authors:** Kennedy Dodam Konlan, Emmanuel Asampong, Phyllis Dako-Gyeke, Franklin N. Glozah

**Affiliations:** 1 Department of Adult Health, School of Nursing and Midwifery, University of Ghana, Legon, Accra, Ghana; 2 Department of Social and Behavioural Sciences, School of Public Health, University of Ghana, Legon, Accra, Ghana

**Keywords:** Allostatic load, Burnout syndrome, Health workers, Ghana

## Abstract

**Background:**

Burnout syndrome is a psycho-social disorder which develops in an individual exposed to chronic stress on the job. Health workers in sub-Saharan Africa (SSA) are at increased risk of burnout due to job-related challenges. Burnout does not only affect the job performance of employees, but could result in dysregulation of multiple physiological systems (allostatic load) in victims and predispose them to non-communicable diseases (NCDs). This study examined the association between burnout and allostatic load among health workers engaged in human resourced-constrained hospitals in Accra, Ghana.

**Method:**

This study was a hospital-based cross-sectional study involving 1264 health workers (clinicians and non-clinicians) from three public hospitals in Accra, Ghana who were recruited using a proportionate stratified random sampling technique. The participants completed a questionnaire which collected general and burnout information. In addition, each participant’s anthropometric; biochemical and hemodynamic indices were measured. The allostatic load in the participants was determined using eleven (11) biomarkers from the neuro-endocrine, cardiovascular, metabolic and anthropometric measures. The relationship between burnout and allostatic overload (high allostatic load) was determined at the bivariate and multivariable levels. The data analysis was done with the aid of Stata 15.0 at a 95% confidence level.

**Results:**

The prevalence of burnout was 20.57%, higher in non-clinicians than clinicians (26.74% vs 15.64, *p* <  0.001). Also, non-clinical participants had higher levels of emotional exhaustion and depersonalization than the clinical participants. Over a quarter (26.27%) of the participants had allostatic overload manifesting as high allostatic load. Furthermore, for a one unit increase in overall burnout, the odds of experiencing allostatic overload was increased by 17.59 times (AOR = 17.59, 95% CI: 11.7-26.4) as compared to those without burnout and similar findings were found for the individual components of burnout syndrome with high allostatic load.

**Conclusion:**

Burnout among health workers is associated with multi-system physiological dysregulation manifesting as high allostatic load; a major risk factor for NCDs. It is recommended that measures aimed at reducing burnout and allostatic overload such as structured psychological counseling and healthy lifestyle patterns are recommended for health workers engaged in stressful work settings to reduce their risk of NCDs.

## Introduction

Health workers are individuals who are engaged in providing health information for promoting healthy lifestyles [1, 2]. They are engaged in actions with the primary intent of preventing diseases [3]. Health workers often deliver health care in teams to promote the health of patients and the welfare of the general public [2]. This places high job demands on each team member and exposes them to job-related burnout [2, 4]. This is further compounded in SSA where health workers are engaged in one of the most challenging work environment due to the limited material and human resources for work [5]. The corona virus disease 2019 (COVID-19) pandemic has further aggravated the workload on the few health workers in SSA [4] predisposing them to additional job-related stress and its associated burnout [6]. In Ghana, there are inadequate material and human resources for the health care system [1, 6] and this compounded by the corona virus disease 2019 [1]. The number of individuals diagnosed with COVID-19 in the Republic of Ghana rose from two (2) positive cases in March, 2020 to over five thousand cases (51667) with over three hundred (323) deaths as at the end of November 2020 [1, 6]. Hospital staff in Accra have therefore been compelled to attend to many individuals especially with the rise in positive COVID-19 cases in 2020 [1, 6] and this predisposes health workers in Accra to high workload and possible associated job-related burnout [1]. Burnout syndrome is a psycho-social disorder resulting from exposure to chronic interpersonal stress at the workplace and characterized by three elements; emotional exhaustion, negative attitude towards service recipients and a feeling of low self-accomplishment [1–7].

Aside burnout affecting the productivity of health workers [2, 4], it is suggested to be a psycho-social risk factor for NCDs among working populations elsewhere [8–10]. The literature [10–12] suggests that burnout contributes to reducing the number of skilled health manpower through NCD-associated mortalities via dysregulation of multiple physiological systems (allostatic load) in victims [13]. According to Sterling and Eyer [14], allostasis describes the mechanism by which physiological stability is achieved through changing processes of bodily systems following exposure to chronic stressors. The allostatic process provides that one of the mechanisms of healthy adaptation to chronic environmental demands is through variability of physiological systems [15]. However, long term variability results in a load being placed on physiological systems; an allostatic load, [15, 16] and this overtime leads to pathologies such as NCDs [17].

Allostasis describes the adjustment of human physiological systems to physical, psychosocial and environmental stressors [8–10]. According to [8], allostasis describes the mechanism by which physiological stability is achieved through changing processes of bodily systems following exposure to chronic stressors. Chronic stress manifesting as burnout has a significant physiological impact on the body of victims [9, 10]. The stress response in itself does not lead to adverse health outcomes; it actually protects the organism from harmful stimuli [8]. However, each time the stress response is activated, physiological adjustments, must be made and over time these adjustments lead to accumulated wear and tear of physiological systems; an allostatic load [8–10]. The concept of allostatic load describes the accumulated wear and tear on person’s physiological systems on exposure to chronic stress; sub-clinical physiological dysregulation [8]. Allostatic load is assessed in physiological systems as imbalances in cardiovascular, metabolic, neuro-endocrine and immune system activity as well as plasticity changes in the brain structures [8]. McEwen and Gianaros [8] identified a number of physiological indicators for determining allostatic load. These included systolic and diastolic BPs, high-density lipoproteins (HDL) and total cholesterol (TC), fasting plasma glucose, glycosylated hemoglobin (HbA1c), levels of plasma glucose, serum dihydroepiandrosterone (DHEA-S), plasma cortisol or 17- Hydroxycorticosteroids or 24-hour urinary cortisol excretion, overnight urinary noradrenaline, adrenalin excretions, BMI and waist circumference.

Most studies on burnout among health workers in SSA [1–10] have largely focused on the impact of burnout on employee attitudes and work output. However, there is a paucity of data on the relationship between burnout and multi-system physiological dysregulation among health workers in Ghana.

### Aim

This study examined burnout and allostatic load among health workers engaged in human resourced-constrained hospitals in Accra, Ghana.

## Methods

### Study design

In this study, we used a cross-sectional study design in which quantitative data was collected from health workers in three hospitals in Accra. The hospitals were purposively chosen to represent the three levels of the public health care system; primary, secondary and tertiary levels [2].

### Setting

The study was conducted in three public hospitals in Accra in the Greater Accra Region of Ghana. Accra is the capital and largest city of Ghana, with a total population of over four million (4,010,054) [2, 18]. As the commercial and political capital of Ghana, Accra receives visitors from all the regions of Ghana particularly; the Central, Volta, Western, Eastern and Ashanti Regions on a daily basis as well as from neighbouring countries such as Burkina Faso, Mali, Niger, la Cote D‟Ivoire, Togo and Nigeria [18]. The high commercial activities and the associated high cost of living place huge demands on the residents of Accra [2]. In addition, health workers in both public and private hospitals in Accra spend several hours to and from work due to heavy vehicular traffic on the roads of the city [2]. These factors predispose them to stress [19] and possible burnout syndrome.

Public hospitals in Accra have higher numbers of patients as compared to the private facilities [18, 20, 21]. This study therefore involved only health workers in the public health facilities who were at increased risk of burnout due to their high patient numbers [22, 23]. The situation is exacerbated by the emergence of COVID-19 pandemic, whereby Accra reportedly has the highest number of COVID-19 cases [6] and thus predisposing health workers in public hospitals in the city to increased workload and job-related burnout [2, 4, 6].

Three public hospitals in Accra were purposively chosen to represent the three levels of the public health care system; primary, secondary and tertiary. The selected hospitals were the Weija-Gbawe Municipal Hospital (WGMH), the Greater Accra Regional Hospital (GARH) and the Korle Bu Teaching Hospital representing the primary, secondary and tertiary levels respectively. The primary level hospital was chosen because of the fact that it had been ranked as the best performing hospital among its peers using a peer ranking system established by the GHS [20]. The secondary level hospital receives referral cases from the district and sub-district health facilities and it is the main secondary level health facility in the Greater Accra Region and serves as the regional hospital and the tertiary level hospital was the national referral hospital and a teaching hospital of the Greater Accra Region [24].

#### Sampling of participants

Health workers in the three chosen public hospitals with not less than 1 year working experience formed the population for the study. The sampling technique that was used for this study was a proportionate stratified random sampling technique. This was done to prevent over representation of a any group or section of HWs over others. A proportionate stratified random sampling is a probability sampling technique in which the researcher divides the population into different homogeneous sub-groups and then randomly selects the final subjects proportionally from the different sub-groups based on their estimated proportionate distribution in the entire population. In selecting the participants, sampling proportionate to size was used to determine the number of health workers recruited in both clinical and non-clinical categories of staff and from the three selected hospitals. This sampling method improves the precision of the sample by reducing the sampling error and also prevents over representation of one homogenous unit in the final chosen sample. The sample size for the study was determined using the sample size determination table designed by Krejcie and Morgan [25].

**Fig. 1:**
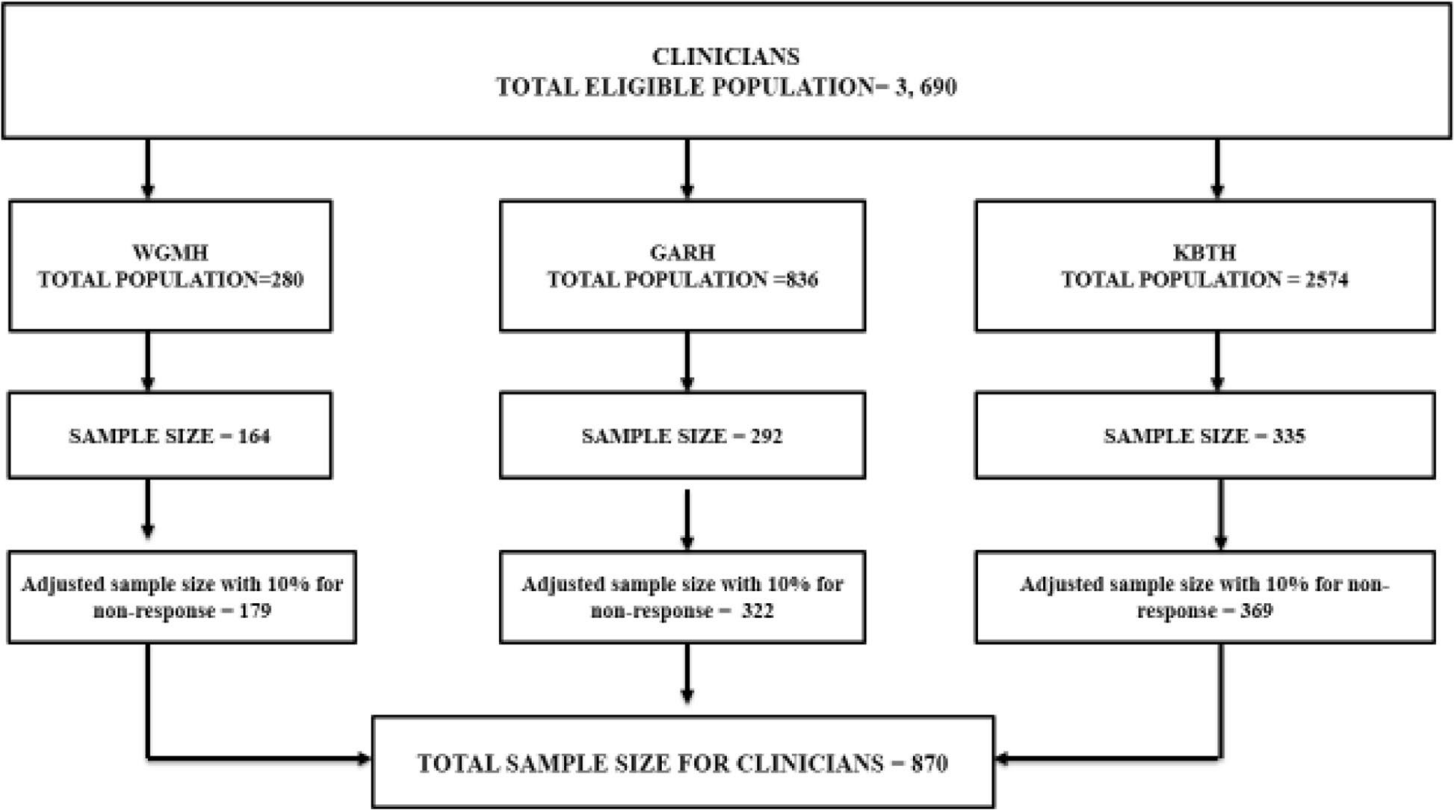
Sample size for clinicians

The total population of eligible clinical staff was three thousand six hundred and ninety (3690) based on the staff distributions for the three hospitals; 280, 836 and 2574 for primary, secondary and tertiary level hospitals respectively as obtained from the human resources (HR) unit of the three hospitals. Using the Krejcie & Morgan [25] tables for sample size determination, these populations of eligible clinical staff corresponded to sample sizes of 162, 292 and 335 participants for the primary, secondary and tertiary level hospitals respectively. After adding 10% for non-response to the sample sizes, these corresponded to the adjusted sample sizes of 179, 322 and 369 participants for the primary, secondary and tertiary level hospitals respectively. Thus, sample size of 870 clinical staff was recruited for the study and this is illustrated in the flow chart below (Fig. 1).

**Fig. 2:**
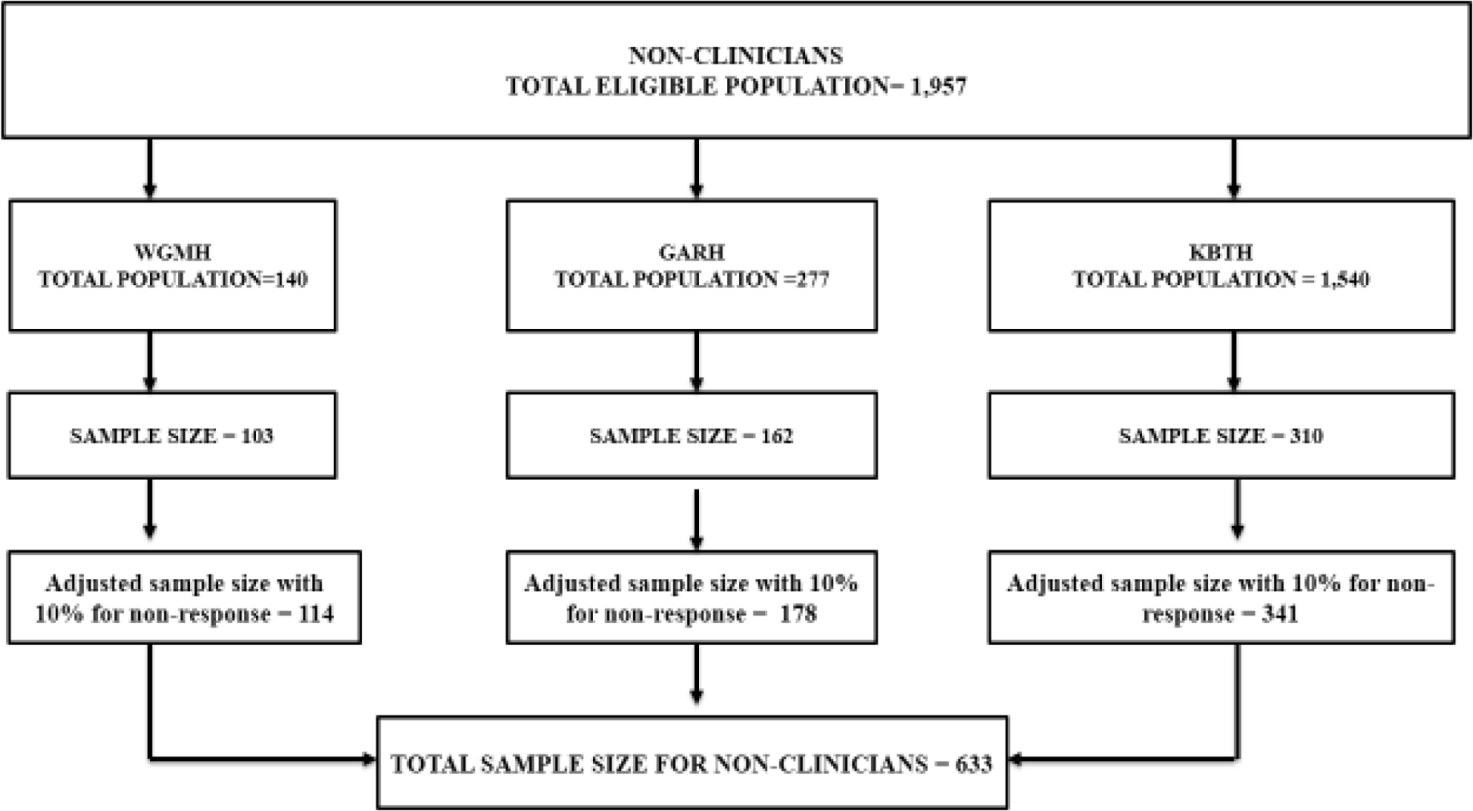
Sample size for non-clinicians

For the non-clinical staff, the total eligible population was one thousand nine hundred and fifty seven (1957) based on the staff distribution for the three hospitals which was 140, 277 and 1540 for primary, secondary and tertiary level hospitals respectively. Using the Krejcie & Morgan [25] table for sample size determination, these populations corresponded to sample sizes of 103, 162 and 310 participants for the primary, secondary and tertiary level hospitals respectively. After adding 10% for non-response to the sample sizes, these corresponded to the adjusted sample size of 114, 178 and 341 participants for the primary, secondary and tertiary level hospitals respectively. Thus, a total sample size of 633 non-clinical staff was recruited for the study (Fig. 2).

Finally, the individual participants were then selected by simple random selection technique of balloting without replacement by the researchers from the sample of clinicians and non-clinicians. Procedurally, a list of all eligible clinicians and non-clinicians were obtained from the HR units of the three hospitals. The eligible staff were put into strata (each professional group of clinicians and each rank of non-clinicians). All the eligible participants in each stratum (each professional group of clinicians and each rank of non-clinicians) were given unique codes. These unique codes were written on small pieces of papers and the pieces of paper placed into covered containers for each professional group of clinicians and each rank of non-clinicians and the covered containers were then shaken thoroughly. After which, the small pieces of papers were picked out at random from each covered container. The number pieces of paper picked from each container representing each stratum (each professional group of clinicians and each rank of non-clinicians) was determined based on staff distribution as per the Ghana Health Service (GHS) [20]; nurses/midwives (69%), doctors (16.1%), pharmacists/pharmacy technicians/ dispensing assistants (4.4%), biomedical scientists/laboratory assistants (3.9%) and the rest of the clinical grades, that is, audiologists, radiographers, dental clinical assistants, physiotherapists (6.6%) as well as senior staff of 38% and junior staff of 62% for non-clinicians [20]. The staff whose identities corresponded with the codes on the pieces of papers that were picked randomly from each container for each professional group and each rank of non-clinical staff were contacted for written informed consent and recruitment at their various units of work in their various hospitals.

### Data collection

Data collection took place between March to November, 2020. Once selected and written informed consent obtained, the participants were given the study questionnaire by trained research assistants (RA), who had at least a Bachelor of Science in nursing or biomedical sciences, at the rest rooms or conference rooms of their various hospitals. The questionnaire collected socio-demographic information such as age, marital status, parenthood, type of facility, common shift, educational background, highest monthly income among others. Also, the questionnaire assessed the level of resilience using the Brief Resilience scale (BRS). The BRS is a six-item likert scale whose scores are summed into a composite score [1]. Scores ranging from 1.0 to 2.99, 3.0 to 4.30 and 4.31 to 5.0 were categorized into low, normal and high resilience respectively [1]. Further, the questionnaire assessed the level of burnout using a validated tool [1]. We adopted the definition of burnout as provided in literature [1, 4, 5]. Each participant completed and returned the study questionnaire in a sealed brown envelope (which was provided by the investigators) within twenty four hours after they had provided written informed consent. Upon the return of the filled questionnaire within twelve (12) to twenty-four (24) hours and confirmation that the minimum fasting requirement had been observed, participants had their anthropometric, body composition and hemodynamic measures taken at their various departments/units in their various hospitals by the trained research assistants. Further, a five (5) milliliter (mls) sample of each participant’s fasting blood was taken by trained research assistants who had knowledge in venipuncture techniques (laboratory scientists) and analyzed for fasting plasma glucose, cortisol, and lipid profile. All the measurements were done between 6:00 and 9:00 GMT.

In line with the guidelines of the GHS against the COVID-19 pandemic, all the RAs and participants were given a free re-usable face mask at no cost to them to wear during the period of the data collection. Also, “Veronica buckets” with water and soap were provided for hand washing with soap under running water in facilities where there was no access to flowing water. Furthermore, alcohol-based hand sanitizers were provided free of charge for the RAs and participants to use in order to protect them from the deadly COVID-19 virus. Additionally, social distancing (6 ft between people) was maintained as far as possible throughout data collection.

### Key measures

#### Burnout syndrome

Burnout syndrome was assessed using a validated tool for assessing burnout in Ghanaian health workers as reported in literature [1]. This was a modified tool from the widely used Maslach Burnout Inventory Human Services Survey (MBI-HSS) tool for diagnosing burnout among health workers [4, 5]. The tool in this study had 22 items [1]. Each item of the 22-item tool was rated on a seven-point Likert scale that measured how frequently the participants experienced a particular feeling (from 0 for never to 6 for every day). The tool measured the three constructs of burnout: Emotional Exhaustion (EE), using nine (9) items to measure physical and emotional depletion; Depersonalization (DP), using five (5) items to measure negative or cynical feelings about patients; and Personal Accomplishment (PA), using eight (8) items to measure how one perceives one’s own competence.

Participants with high scores on emotional exhaustion and depersonalization as well as low scores on personal accomplishment sub-scales were diagnosed as burnt or said to be experiencing burnout [1, 4, 5, 7].

### Level of resilience

The level of resilience in each respondent was measured using the Brief Resilience scale (BRS). The BRS is a six-item likert scale whose scores were summed into a composite score [1]. Scores ranging from 1.0 to 2.99, 3.0 to 4.30 and 4.31 to 5.0 were categorized into low, normal and high resilience respectively [1].

### Anthropometric measurement

Weight was measured with participants barefooted and wearing light clothing using an Omron digital scale (HN-288), and it was recorded to the nearest 0.1 kg. Height was measured using the Seca Stadiometer (Seca, Germany) with participants in an erect position and barefooted, with shoulders in normal alignment. Body mass index (BMI in kg/m 2) was calculated for each participant as the individual’s body weight (in kilograms) divided by the square of height (in meters). BMI was categorized as underweight (BMI < 18.50 kg/m2), normal weight (BMI: 18.50 – 24.99 kg/m2), overweight (BMI: 25.00 – 29.99 kg/m2) and obese (BMI ≥ 30 kg/m2). In the measurement of waist and hip circumferences, each participant was made to stand with his arms at the sides, feet positioned close together, and weight evenly distributed across the feet. Measurements for waist circumference (WC) were made at the end of a normal expiration, with a non-elastic tape measure, at the approximate midpoint between the lower margin of the last palpable rib and the top of the iliac crest. Hip circumference was measured at the level of the greater trochanters. Waist-hip ratio was determined as the ratio of waist circumference and the circumference of the hip.

### Hemodynamic measurements

Systolic and diastolic BPs were measured using an automated digital BP monitor (Omron 991 XL, Health care, Inc., Vernon Hills, IL). Before the BP measurement, the participants were asked to empty their urinary bladder if they had not passed urine within the last four (4) hours to prevent indirect pressure of a full urinary bladder on the aorta at the aortic bifurcation around the iliac region. The blood pressure cuff was placed on the left arm of the participant lying in a supine position on an examination bed; with the lower edge of the cuff about 2-3 cm above the elbow crease and the bladder centered over the brachial artery. The arm was rested on a table and raised so that the cuff was at the level with the heart. The participants were allowed to rest for at least 5-10 minutes prior to the BP measurements. The blood pressure was measured three times; each measurement was spaced so that it occurred at least a 60 second interval after the preceding. The first measurement was discarded and the last two measurements were averaged to give the BP for each participant.

### Biochemical analysis

After a minimum of 8 hours fasting, 5 mls of venous blood was obtained from antecubital area. The blood was centrifuged and aliquoted. Fasting plasma glucose was measured with a Selectra Junior chemical auto analyzer from the United Kingdom (Bayer Diagnostics, UK), using ELITech glucose PAP SL reagent from ELITech clinical systems, France, following the manufacturer’s instructions. Also, lipid profile which assessed total cholesterol, high density lipoprotein cholesterol (HDL), and triglycerides. The level of low density lipoprotein cholesterol was determined using Friedewald’s formula. The Lipid profile of plasma was analyzed using Selectra Junior chemical auto analyzer from the United Kingdom (Bayer Diagnostics, UK), using ELITech cholesterol SL, ELITech cholesterol HDL SL 2G and ELITech triglycerides Mono SL New reagents from ELITech clinical systems, France, following the manufacturer’s instructions.

Plasma cortisol was assayed using an enzyme linked immunoassay (ELISA) method using cortisol Elisa Kit (Cayman Chemical).

### Definition of allostatic load

The allostatic load in the participants was determined using eleven (11) biomarkers from the neuro-endocrine, cardiovascular, metabolic and anthropometric measures as recommended [16, 26–28]. The biological markers were: cortisol (neuro-endocrine), mean systolic BP, mean diastolic BP and heart rate (cardiovascular system); BMI and waist-to-hip ratio (anthropometric); and HDL, LDL, total cholesterol, triglycerides and fasting plasma glucose (metabolic). Each biological marker was dichotomized into high versus low-risk values (1–0) according to clinical thresholds as found in the literature [16, 27–29]. The total numerical scores of the allostatic load scores for each participant were computed by summing up the dichotomized values, and these ranged between 0 and 11. The final allostatic scores were dichotomized into high versus low allostatic load risk by using the median (5.5) as a cut-off value. Scores at or above the median were seen as high allostatic load (allostatic overload) while those below the median were classified as low allostatic load [28].

### Statistical analysis

Stata 15.0 was used to aid with the analysis. A *p*-value of less than 0.05 was deemed to be significant. Comparisons of general and physiological characteristics was done based on the category of participants (clinicians versus non-clinicians) using frequencies, student t-test for continuous variables and chi-square for categorical variables. Bivariable models were used to predict the associations between burnout and allostatic load. After the binary analysis, multivariable logistic regression was used to determine the strength of association. The multivariable logistic regression was done at two levels. The first was to establish the crude level of association between burnout and allostatic load. In this strategy, all independent variables known to affect allostatic load were added to the first model to establish the crude relationship with the dependent variable at various levels of measurement. Afterwards, variables that were significant in the first model and at the bivariable analysis level were put in a multivariable binary and ordinal logistic regression model and adjusted for age, sex, level of resilience, common shift, and familial history of CVDs.

## Results

### General characteristics of participants

The total number of participants who took part in the study was one thousand two hundred and sixty four health workers (1264) and this represented a response rate of 84.10%. The participants’ mean age was 40.81 ± 8.33 years. More than half of the participants (53.09%) were females with 61.31% being married. The study found that majority of the health workers (72.87%) had tertiary education which was classified as high education level. Similarly, almost half (43.04%) of the participants said they did additional jobs aside their current jobs (Table 1).

**Table 1:** General characteristics of participants

General characteristics	Clinical, ***n*** (%) 703 (55.62)	Non-Clinical, n (%) 561 (44.38)	Total, ***n*** (%) 1264, (100)
**Age in years: mean (± SD)**	41.05 ± 8.30	40.51 ± 8.38	40.81 ± 8.33
**Sex of respondent**			
Male	317 (45.09)	276 (49.20)	593 (46.91)
Female	386 (54.91)	285 (50.80)	671 (53.09)
**Marital status**			
Single	106 (15.01)	99 (17.65)	205 (16.22)
Married	428 (60.88)	347 (61.85)	775 (61.31)
Divorced/Separated	169 (24.04)	115 (20.50)	150 (22.47)
**Having Children**			
Yes	409 (57.75)	399 (71.12)	808 (63.92)
No	294 (41.82)	162 (28.88)	456 (36.08)
**Highest educational level**			
Low	0(0)	33 (5.88)	33 (2.61)
Middle	6 (0.85)	304 (54.19)	310 (24.53)
High	697 (99.15)	224 (39.93)	921 (72.87)
**Range of net monthly income**			
Low	251 (35.70)	483 (37.79)	734 (58.07)
Middle	392 (55.76)	78 (66.13)	470 (37.18)
High	60 (8.53)	0 (0)	60 (4.75)
**Years of working**			
1-5	267 (37.98)	227 (40.46)	494 (39.08)
6-10	133 (18.92)	88 (15.69)	221 (17.48)
11-15	23 (3.27)	50 (8.91)	73 (5.78)
16-20	50 (7.11)	54 (9.63)	104 (0.23)
20	230 (32.72)	142 (25.31)	372 (29.43)
**Level of resilience**			
Low	166 (23.61)	160 (28.52)	326 (25.79)
Normal	271 (38.55)	209 (37.25)	480 (37.97)
High	266 (37.84)	131 (23.35)	458 (36.23)
**Facility**			
Primary	149 (21.19)	134 (23.89)	283 (22.39)
Secondary	264 (37.55)	142 (25.31)	406 (32.12)
Tertiary	290 (41.25)	285 (50.80)	575 (45.49)
**Common shift for past six months**			
Night	269 (38.26)	332 (59.18)	601 (47.55)
Afternoon	228 (32.43)	146 (26.02)	374 (29.59)
Morning	206 (29.30)	83 (14.80)	289 (22.86)
**Additional jobs**			
Yes	339 (48.22)	205 (36.54)	544 (43.04)
No	364 (51.78)	356 (63.46)	720 (56.96)
**Familial History of NCDs**			
Yes	247 (35.14)	199 (35.47)	446 (35.28)
No	456 (64.86)	362 (64.53)	818 (64.72)

Frequency (n) and percentage (%)

### Physiological characteristics of the participants

The mean age of the participants was 40.81 ± 8.33 years. Non-clinicians were taller, had more visceral fat, hip-circumferences, mean plasma cortisol level and HDLs as compared to the clinicians. However, the clinicians had higher weight, BMI, waist circumferences, waist-hip ratios, mean total cholesterol and LDL (Table 2).

**Table 2:** Physiological characteristics of participants

Physiological Indices	Total ***N*** (%) 1264 (100%) (π ± SD)	Clinical (***N*** = 703) (π ± SD)	Non-Clinical (***N*** = 561) π ± SD)	P
**Anthropometry**				
Height, cm	161.77 ± 6.91	160.68 ± 7.11	163.13 ± 6.40	**< 0.001**
Weight, kg	69.06 ± 10.24	69.23 ± 9.89	68.86 ± 10.66	**< 0.001**
BMI	26.46 ± 3.93	26.90 ± 3.98	25.90 ± 3.79	**< 0.001**
Body Fat, %	26.93 ± 0.02	27.24 ± 0.08	26.54 ± 0.03	0.125
Visceral Fat, %	8.50 ± 0.16	8.10 ± 6.00	9.02 ± 5.65	**0.005**
Hip circumference, cm	100.5 ± 15.35	99.40 ± 15.24	101.55 ± 15.34	**0.003**
Waist circumference, cm	93.46 ± 11.54	94.15 ± 11.67	93.46 ± 11.38	0.292
Waist-Hip Ratio (WHR)	0.97 ± 0.19	1.0 ± 0.19	0.95 ± 0.19	**< 0.001**
**Hemodynamic**				
Systolic BP, mmHg	127.32 ± 15.33	128.01 ± 15.33	126.46 ± 15.08	0.07
Diastolic BP, mmHg	78.66 ± 11.07	78.65 ± 11.25	78.68 ± 10.84	0.951
Heart rate, beats/minute	73.78 ± 12.66	72.65 ± 12.79	75.18 ± 12.37	< 0.001
Mean BP, mmHg	95.03 ± 12.047	95.24 ± 12.21	94.75 ± 11.90	0.467
**Biochemical**				
FPG, mmol/l	5.44 ± 1.16	5.49 ± 1.12	5.43 ± 1.21	0.342
Cortisol, nmol/l	441.9 ± 198.3	433.5 ± 195.6	451.7 ± 201.3	0.042
Total Cholesterol, mmol/l	5.24 ± 1.3	5.34 ± 1.29	5.05 ± 1.20	0.020
Triglycerides, mmol/l	1.68 ± 020	1.68 ± 0.21	1.67 ± 0.19	0.22
HDL, mmol/l	0.92 ± 0.24	0.91 ± 0.24	0.92 ± 0.25	0.491
LDL, mmol/l	3.25 ± 0.95	3.31 ± 0.87	3.18 ± 1.03	**0.025**

Data are presented as mean (± standard deviation), *p* values were determined using t tests

### Prevalence of burnout and high allostatic load (allostatic overload)

The prevalence of burnout in our study was 20.57% with non-clinicians showing higher prevalence of burnout as compared to clinicians. Also, 26.27% of the participantsparticipantsparticipants had high allostatic load (Table 3).

**Table 3:** Prevalence of burnout and allostatic load among participants

	Total ***N*** (%) 1264 (100%)	Clinical (***N*** = 703)	Non-Clinical (***N*** = 561)
**Burnout**			
Yes	260 (20.57)	110 (15.65)	150 (26.74)
No	1004 (79.43)	593 (84.35)	411 (73.26)
**Allostatic load**			
High	332 (26.27)	184 (26.17)	148 (26.38)
Low	932 (73.73)	519 (73.83)	413 (73.62)

Frequency (n) and percentage (%)

### Association between burnout and allostatic load

High emotional exhaustion was found to be significantly associated with high allostatic load. Similarly, high depersonalization and low personal accomplishment were found to be significantly associated with high allostatic load. Also, overall burnout was associated with high allostatic load among the participants (Table 4).

**Table 4:** Association between burnout and high allostatic load and burnout

Components of burnout	High Allostatic load ***N*** (%) 332 (100)	***X***^***2***^, ***p***-value
**Emotional Exhaustion**		409.4, **< 0.001**
Low	46 (13.85)	
Moderate	26 (7.83)	
High	260 (78.31)	
**Depersonalization**		293.6, **< 0.001**
Low	112 (33.73)	
Moderate	30 (9.03)	
High	190 (57.23)	
**Personal Accomplishment**		159.4, **< 0.001**
Low	207 (62.34)	
Moderate	72 (21.69)	
High	53 (15.96)	
**Burnout**		340.6, **< 0.001**
Yes	185 (55.72)	
No	147 (44.28)	

Among the study participants, high emotional exhaustion, high depersonalization and low personal accomplishment were associated with increased odds of experiencing high allostatic load in unadjusted logistic regression models. After adjustment for the general and physiological characteristics of participants associated with high allostatic load; overall burnout, high emotional exhaustion and high depersonalization were associated with increased odds of experiencing high allostatic load as compared with those with no or low burnout. Specifically, the results showed that participants with overall burnout were 17.59 times more likely (AOR = 17.59, 95% CI = 11.7- 26.4) to experience high allostatic load as compared those without burnout. Also, participants with high emotional exhaustion were 17.90 times more likely (AOR = 17.90, 95% CI = 11.6- 27.8) to experience high allostatic load as compared those without high emotional exhaustion. Similarly, participants with high depersonalization were 9.74 times more likely (AOR = 9.74, 95% CI = 6.90- 13.76) to experience high allostatic load as compared those without high depersonalization. Likewise, participants with low personal accomplishment were 1.10 times more likely (AOR = 1.10, 95% CI = 0.74- 1.64) to experience high allostatic load as compared those without low personal accomplishment (Table 5).

**Table 5:** Multivariate logistic regression of the relationship between high allostatic load and burnout

Independent Variables	High Allostatic load					
	Crude Odd ratio	95% CI	***P***-value	Adjusted Odd ratio	95% CI	***P***-value
**Burnout**						
No	**Reference**					
Yes	14.38	10.44-19.81	< 0.001	17.59	11.7-26.4	**< 0.001**
**Emotional Exhaustion**						
Low	**Reference**					
Moderate	0.71	0.43-1.17	0.103	0.78	0.47-1.31	0.355
High	14.97	10.42-21.94	< 0.001	17.90	11.57-27.8	**< 0.001**
**Depersonalization**						
Low	**Reference**					
Moderate	6.95	4.03-11.96	< 0.001	5.94	3.34-10.58	**< 0.001**
High	10.64	7.88-14.38	< 0.001	9.74	6.90-13.76	**< 0.001**
**Personal Accomplishment**						
Low	0.67	0.48-0.94	0.021	1.10	0.74-1.64	0.643
Moderate	0.14	0.10-0.19	< 0.001	0.18	0.12-0.26	< **0.001**
	**Reference**					

Logistic age, sex, level of resilience, common shift, and familial history of CVDs

## Discussion

The study found that more than half of the participants were females. This is in line with reports from other studies [3, 19, 30] that females constitute the majority of the health workforce in SSA. However, Pindar and Coker [31] found males to be the dominant health workers in northern Nigeria. The high number of females among the participants in this study was because nurses and midwives who form the majority of the health workforce are predominantly females [19, 32, 33]. In addition, the results revealed that majority of the health workers were clinicians and this is in consonance with other studies in Ghana [2–4, 19] that have established that clinicians are the dominant category of hospital staff.

The finding of high prevalence of burnout in this study is in consonance with the findings of Asiedu et al. [19] who reported high burnout levels among nurses in Ghana. Similarly, Afulani et al. [4] reports that burnout is high among Ghanaian health workers and that inadequate preparedness towards the COVID-19 pandemic further compounds the situation. The fear of contracting the deadly virus makes health workers feel stressed-out and developed burnout eventually. Also, the findings in this study are in line with those of Afulani et al. [8] that almost 30% of health workers in Kenya have burnout. In addition, the findings are in line with those found by He et al. [9] in which burnout was reported to be high among health workers in China. However, the prevalence of burnout in this study is higher than the little over 10 % found by Habadi et al. [34] among Saudi Arabian health professionals. Likewise, the finding in this study is higher than the less than the ten (10) percent prevalence found by Ayisi-Boateng et al. [22], Yunus et al. [35] and [2] among some sections of health workers. Similarly, the findings are in contrast with those of Opoku and colleague among physicians in which burnout levels was found to be low [36]. Furthermore, the findings are in contrast with what Langade et al. [37] observed among health workers in India where they reported low prevalence of burnout.

This finding of high overall burnout among the participants could be because of the inadequate resources for health care as reported by earlier studies in Ghana [2, 19, 22, 36, 38]. Also, the rising cost of health care more especially with the emergence of COVID-19 [6] as well as the fear of contracting the deadly virus in the line of duty by health workers could be implicated in the high burnout [4].

The study found that overall burnout as well as the individual components of burnout syndrome was significantly associated with physiological dysregulation manifesting as high allostatic load. This finding is supported by other studies [8–13, 26–28] that have pointed to the dilapidating effects of job-related burnout on the physiology of victims. It is suggested that burnout could lead to NCDs particularly cardiovascular diseases through dysregulation of the activity of the heart and blood vessels as well as poor metabolism of fat [8–11]. The literature [26–28, 33] states that chronic stress manifesting as burnout has significant physiological impact on the body of victims and that this is often negative due to its longer duration. Whenever the stress response is activated, physiological adjustments, must be made and over time these adjustments lead to accumulated wear and tear of physiological systems; an allostatic load [13–17] and that exposure to stress for longer duration, causes sub-clinical dysfunction of physiological systems of victims leading to chronic diseases [16, 26–28]. Other studies [9–12] have pointed to this allostatic load pathway as the plausible link between job-related burnout and NCDs among employees engaged in stressful job-settings.

Some authors [8–13, 27, 28] cite chronic stress hormonal dysregulation particularly dysfunctional negative feedback loop from the hypothalamo-hypophyseal (pituitary)-adrenocortical system to be principally responsible for this burnout induced physiological dysregulation. They aver that chronic stress leads to a surge in glucocorticoid hormones (cortisol) and this tend to affect the normal functions of the heart, blood vessels as well as glucose and fat metabolism. This thus predisposes individuals with this hypercortisolemia to NCDs. Other authors [9, 11, 33] have argued that the protective effect of estrogen is lost in female employees experiencing burnout syndrome and this contributes in enhancing allostatic overload [26–28].

Some authors [9, 11, 33] have further suggested that burnout triggers unhealthy lifestyles such as poor dietary patterns, alcohol use, cigarette smoking among others in employees due to its associated depressive symptoms and these could predispose employees experiencing burnout to dysregulation of most of their physiological systems as these unhealthy lifestyle choices are themselves independent risk factors for physiological dysregulation as well as chronic diseases.

Essentially, the physiological dysregulation in employees experiencing burnout is multi-faceted; ranging from hormonal dysregulation through to unhealthy lifestyle choices and therefore complex to resolve once an individual experiences burnout. The combination of these factors contributes to increasing the risk of NCDs among individuals experiencing burnout and this is a major threat to public health particularly if health workers who ought to be role models for the public become victims.

## Limitations of the study

This was a cross-sectional observational/non-experimental study, thus, associations described are not causal. However, associations observed from this study provide some basis for the use of other study designs to establish causation.

One of the limitations of this study is that each biological marker is divided into high and low risk values, which is still an experimental device. More studies are needed to help conceptualize allostatic load. Similarly, some authors who have used the MBI (Maslach and Leiter [39]) have always been reluctant to indicate cut-off points for each of the dimensions of burnout, measured with this method. Rather, they will point out that they must be taken precisely as “dimensions” and that it is impossible to establish cut-off points but other studies [1–6] have suggested the dimensions can be put together to diagnose burnout as a condition.

Also, the study only depicts a relationship between the variables and does not in any way infer causation. However, the findings provide insights for possible longitudinal and experimental studies to establish possible causation.

In addition, per the sample size calculation, the overall minimum sample size for clinicians and non-clinicians was 1366. However, the number of study participants who completed the study was 1264. This is less than the minimum sample size required for the study. Hence the study was under-powered and the scientific rigor compromised. However, the study sets a stage on what percentage of the minimum sample size should be added to the sample size to cater for non-response. It is recommended that future studies among health workers consider a non-response rate of 20 % (20%) as compared to the 10% non-response rate that was added to the sample size in this study.

Furthermore, the data collection was done in the mornings between 6:00 am to 9:00 am due to the biochemical parameters like fasting plasma glucose, cortisol and lipid profile which are best measured from morning blood samples. This meant that majority of those who took part in the study were either night or morning duty staff who were largely at post during the time of the data collection. However, to overcome this challenge, the researcher recruited most of the participants in the evenings a day before their involvement in the study.

## Conclusion

Burnout among health workers is associated with dysregulation of multiple physiological systems of victims manifesting as allostatic overload and these could result in future NCDs. It is recommended that measures aimed at reducing burnout and allostatic overload such as structured psychological counseling (mentorship and peer-peer counselling) and healthy lifestyle patterns such as structured exercise regimen are recommended for health workers engaged in stressful work settings to reduce their risk of NCDs. in order to promote the health and wellbeing of health workers so as to meet the sustainable development goal targets related to health.

## Data Availability

The datasets generated and/or analyzed during the current study are not publicly available yet as some aspect of the data is been analysed for other manuscripts but are available from the corresponding author on reasonable request.

## Abbreviations

GHS: Ghana Health Service
GARH: Greater Accra Regional Hospital
HR: Human Resources
KBTH: Korle Bu Teaching Hospital
NCDs: Non-Communicable Diseases
WGMH: Weija-Gbawe Municipal Hospital
